# Screening of MMP-13 Inhibitors Using a GelMA-Alginate Interpenetrating Network Hydrogel-Based Model Mimicking Cytokine-Induced Key Features of Osteoarthritis In Vitro

**DOI:** 10.3390/polym16111572

**Published:** 2024-06-01

**Authors:** Qichan Hu, Steven L. Williams, Alessandra Palladino, Melanie Ecker

**Affiliations:** 1 Department of Biomedical Engineering, University of North Texas, Denton, TX 76203, USA; 2 Department of Biological Sciences, University of North Texas, Denton, TX 76203, USA

**Keywords:** IPN hydrogel, GelMA, sodium alginate, MMP-13 inhibitors, drug screening, cartilage explants

## Abstract

Osteoarthritis (OA) is a chronic joint disease characterized by irreversible cartilage degradation. Current clinical treatment options lack effective pharmaceutical interventions targeting the disease’s root causes. MMP (matrix metalloproteinase) inhibitors represent a new approach to slowing OA progression by addressing cartilage degradation mechanisms. However, very few drugs within this class are in preclinical or clinical trial phases. Hydrogel-based 3D in vitro models have shown promise as preclinical testing platforms due to their resemblance to native extracellular matrix (ECM), abundant availability, and ease of use. Metalloproteinase-13 (MMP-13) is thought to be a major contributor to the degradation of articular cartilage in OA by aggressively breaking down type II collagen. This study focused on testing MMP-13 inhibitors using a GelMA-alginate hydrogel-based OA model induced by cytokines interleukin-1 beta (IL-1β) and tumor necrosis factor alpha (TNF-α). The results demonstrate a significant inhibition of type II collagen breakdown by measuring C2C concentration using ELISA after treatment with MMP-13 inhibitors. However, inconsistencies in human cartilage explant samples led to inconclusive results. Nonetheless, the study highlights the GelMA-alginate hydrogel-based OA model as an alternative to human-sourced cartilage explants for in vitro drug screening.

## 1. Introduction

Osteoarthritis (OA) is the most common form of arthritis, characterized by the progressive breakdown of cartilage in the joints. Cartilage is tough, flexible connective tissue encasing the terminal ends of bones at a joint and helps facilitate smooth movement. As cartilage deteriorates, affected bones may begin to rub against each other, producing pain, stiffness, impaired joint function, and reduced range of motion. OA is a multifactorial disease influenced by a combination of genetic, environmental, and lifestyle factors [1]. Currently, there is no cure for OA; however, various treatment and management strategies can help alleviate symptoms, improve joint function, and enhance quality of life for OA patients. These treatments include medications, lifestyle modifications, physical therapy, surgical intervention, and assistive devices [2]. 

In recent years, novel classes of disease-modifying osteoarthritis drugs (DMOADs) have been developed, such as sprifermin [3], strontium ranelate [4], cathepsin K inhibitors [5], and MMP (matrix metalloproteinase) inhibitors [6]. Unlike conventional OA treatments, primarily involving symptom management, DMOADs aim to modulate the underlying pathophysiology of OA to preserve joint structure and function over time by slowing cartilage degradation. MMP-13 is a key enzyme involved in the degradation of type II collagen, which is the primary structural component of articular cartilage. Increased MMP-13 activity has been implicated in the pathogenesis of OA, as it leads to the excessive degradation of type II collagen, contributing to cartilage breakdown and irreversible joint damage [7]. Therefore, clinical interest in targeting MMP-13 activity and inhibiting its functionality has gained traction and resulted in the development of novel MMP-13 inhibitors.

Preclinical studies have provided strong evidence supporting the role of MMP-13 in OA pathogenesis. It has been evaluated in both animal models and cartilage explants, demonstrating the efficacy of MMP-13 inhibitors in reducing cartilage degradation and slowing disease progression, thereby laying the foundation for further development and clinical testing [8,9]. However, despite the significant focus on MMP-13 inhibitors, few drugs are reported as being in human clinical trials according to publicly available databases at the time of this publication. This reflects the need for further MMP-13 inhibitor research in an effort to translate this drug class’s preclinical promise into an effective pharmaceutical option for OA management.

Every type of disease model has its own advantages and disadvantages. Live animal models offer a more contextual representation of disease pathophysiology in multicellular organisms by replicating complex interactions between cell types, tissues, and organ systems. However, due to genotypic and phenotypic differences between human and non-human species, animal trial results may be too general or non-translatable. Live tissue explant cultures provide a physiologically representative model with native architecture and cell–cell interactions, allowing for tissue-level responses to drugs. Yet, heterogeneity among explants can affect reproducibility. Hydrogel-based models mimic human tissue and disease microenvironments, offering a species-specific insight into drug responses without the uncertainty of interspecies compatibility inherit to animal models. These models enable high-throughput drug screening, thereby allowing researchers to evaluate large compound libraries or simultaneously test multiple experimental conditions. Therefore, complementing animal model studies and other experimental approaches with hydrogel models may further validate preclinical findings.

GelMA (gelatin methacryloyl) is a photocrosslinkable hydrogel derived from gelatin, offering excellent biocompatibility and tunable mechanical properties that closely resembles natural ECM found in human cartilage. Its ability to support cell adhesion, proliferation, and differentiation makes it an ideal candidate for 3D cell culture and tissue engineering applications [10]. Additionally, sodium alginate (SA) is a biocompatible polysaccharide derived from seaweed, known for its superior water retention capacity and gel-forming ability under mild conditions [11,12]. When combined with GelMA, sodium alginate can enhance the stability and mechanical strength of the hybrid hydrogel while maintaining a hydrated microenvironment conducive to cell survival and ECM synthesis [12]. Additionally, sodium alginate serves as an effective reservoir for growth factors or therapeutic agents, enabling the sustained release and localized delivery of compounds within the hydrogel matrix [13]. The synergistic properties of GelMA and sodium alginate are ideally suited for cartilage tissue engineering, providing a biomimetic environment with customizable mechanical properties, high water retention, and support for cell encapsulation and viability.

In this study, we focused on the development of cartilage constructs supported by a GelMA-alginate interpenetrating polymer network (IPN) hydrogel scaffold. By loading this hydrogel formulation with chondrocytes, we sought to create a biomimetic OA model that closely resembles the native cartilage environment, which facilitates the screening of MMP-13 inhibitors for their potential therapeutic efficacy.

## 2. Materials and Methods

### 2.1. Synthesis and Characterization GelMA-Alginate IPN Hydrogel 

#### 2.1.1. Synthesis of GelMA-Alginate IPN Hydrogel

The pre-polymer GelMA was synthesized according to our established protocol [14]. Briefly, 10% (*w*/*v*) type B gelatin (~225 g Bloom, Sigma-Aldrich, St. Louis, MO, USA) dissolved in 0.25 M carbonate-bicarbonate buffer (pH 9.0) was reacted with methacrylic anhydride (Sigma-Aldrich, St. Louis, MO, USA) at the ratio of 1:0.6 (g/mL) and dialyzed against distilled water with Spectrum™ Spectra/Por™ 2 RC Dialysis Membrane Tubing (molecular weight cutoff 12–14 kDa) for 120 h at 40 °C to remove the unreacted groups. The dialyzed GelMA solution was then lyophilized in a freeze dryer (Labconco, Kansas, MO, USA) for 96 h, generating a porous white foam, then stored at −80 °C. GelMA-alginate hydrogel was prepared by photopolymerization and ionic polymerization of two prepolymers—GelMA and sodium alginate (Sigma-Aldrich, St. Louis, MO, USA)—mixed at 1:1 ratio in an aqueous solution. First, a 15% (*w*/*v*) GelMA solution was prepared by dissolving the lyophilized GelMA in a 0.05% lithium phenyl-2,4,6-trimethylbenzoylphosphinate (LAP) photoinitiator (Sigma-Aldrich, St. Louis, MO, USA) PBS solution under heat at 37 °C for 30 min. A total of 1, 2, 3, and 4% (*w*/*v*) sodium alginate solutions were prepared by dissolving the required amounts for each in PBS under continuous stirring overnight at room temperature. Then, equal volumes of GelMA and sodium alginate solution were mixed by pipette and kept at 37 °C in a water bath to allow the GelMA and sodium alginate mixture to fully blend. Finally, hydrogels were cast in a 24-well plate with each well containing 1 mL of the GelMA-sodium alginate solution, followed by exposure to 365 nm UV light for 2 min, and subsequent immersion in 100 mM CaCl_2_ for 10 min to complete polymerization.

#### 2.1.2. Microstructure of GelMA-Alginate IPN Hydrogel

The cross-sectional morphology of the hydrogels was examined using scanning electron microscopy (SEM TM3030Plus, Hitachi, Tokyo, Japan), operated at 15 kV, following lyophilization and carbon spraying.

#### 2.1.3. Compressive Modulus of GelMA-Alginate IPN Hydrogel

The compressive modulus was measured using a MicroTester G2 (CellScale, Waterloo, ON, Canada), according to a modified ASTM D695 protocol. Cylindrical samples measuring 4 mm × 4 mm were prepared with a biopsy punch. Before testing, the samples were immersed in PBS at room temperature for 24 h to reach swelling equilibrium. During testing, a tungsten microbeam (1.5748 mm diameter) was affixed to a vertical actuator on one end and 5 × 5 mm² compression platen on the other to compress the hydrogel samples. Displacement of the compression platen was tracked using a camera, while the gel was compressed to a final strain of 5% within 20 s, held for 5 s, and allowed to recover for 20 s. The compressive modulus was determined from the slope of the stress–strain curve’s linear region. 

#### 2.1.4. Swelling Degree of GelMA-Alginate IPN Hydrogel 

The swelling degree of the hydrogels was assessed based on water absorption of the freeze-dried samples. To begin, freshly prepared hydrogels were washed for 5–10 min in PBS, lyophilized for 24 h, then weighed to determine the mass of the crosslinked hydrogels (*W*_d_). After rehydrating in PBS at room temperature for 48 h, the gels were re-weighed (*W*_s_). The swelling degree was then calculated using the following equation [15]:

Swelling degree = [(*W*_s_ − *W*_d_)/*W*_d_] × 100%

### 2.2. Cell Viability in GelMA-Alginate IPN Hydrogels

#### 2.2.1. Cell Culture and Encapsulation

Human chondrocytes TC28a2, kindly provided by Dr. Miguel Otero from the HSS Research Institute, were cultured in complete culture medium (CCM) composed of DMEM/F12 media (ATCC) supplemented with 10% FBS and 100 units/mL of Penicillin–Streptomycin (Gibco, Thermo Fisher Scientific, Waltham, MA, USA). At 80–90% confluency, the cells were detached using a 0.25% Trypsin-EDTA solution and counted using a Countess II Cell Counter (Invitrogen, Thermo Fisher Scientific, Waltham, MA, USA). The GelMA-sodium alginate solution was prepared as described in Section 2.1.1 and sterilized with a 0.22 µm filter. The solution was then warmed to 37 °C and mixed with the cultured TC28a2 chondrocytes at a density of 1 × 10^7^/mL with gentle, thorough pipetting to ensure mixture homogeneity. The complete cell-laden hydrogels were formed by dispensing 40 µL of the mixture into each well of a 24-well plate, followed by exposure to UV light for 2 min, then immersed in 37 °C (pre-warmed) 100 mM CaCl_2_ for 10 min. Afterward, the newly formed hydrogel discs were transferred to a new 24-well plate, one disc per well, with a fresh-medium change every 3 d. After initial formation, the cell-laden hydrogel discs were sampled on days 1 and 7 for cell viability testing.

#### 2.2.2. Live/Dead Staining

The viability of the chondrocytes within the hydrogel matrix was evaluated using the LIVE/DEAD™ Viability/Cytotoxicity Kit (Invitrogen, Thermo Fisher Scientific, Waltham, MA, USA), according to the manufacturer’s instructions. The cell-laden hydrogels were washed 2× with PBS for 15 min and incubated with 300 µL of staining solution (2 μM calcein-AM and 4 μM EthD-1 in PBS) in each well at 37 °C for 30 min. Afterward, the staining solution was removed and the samples were again washed with PBS and imaged through a fluorescence microscope (Keyence, Des Peres, MO, USA). The images were analyzed using ImageJ (version 1.54 g).

#### 2.2.3. AlamarBlue™ Assay

Cell viability in hydrogels was assessed using the alamarBlue™ (Thermo Fisher Scientific, Waltham, MA, USA) assay according to the manufacturer’s instructions. The culture medium was replaced with fresh medium containing 10% alamarBlue™ reagent. This reagent contains a cell-permeable dye that is reduced by metabolically active cells, producing a fluorescent signal proportional to cell viability. The hydrogel samples were incubated with the alamarBlue™ reagent for 24 h at 37 °C, allowing the dye to penetrate the hydrogel and interact with viable cells. The fluorescence intensity (FI) was measured using a microplate reader (Bio Tek, Winooski, VT, USA) and cell viability was quantified based on the level of fluorescence emitted by the reduced dye.

### 2.3. Chondrogenic Differentiation 

#### 2.3.1. Optimization of Chondrogenic Differentiation Medium in 2D Culture

The chondrocytes were seeded in 24-well plates at a density of 5 × 10^4^ cells/well in CCM. After a monolayer formed overnight, the cells were divided into four groups: (1) CCM + 50 μg/mL L-ascorbic acid 2-phosphate (AA2P) + 40 ng/mL Dexamethasone (DXM); (2) CCM + 50 μg/mL AA2P + 40 ng/mL DXM + 10 ng/mL transforming growth factor beta 1 (TGF-β1); (3) CCM + 50 μg/mL AA2P + 40 ng/mL DXM + 10 ng/mL TGF-β1 + ITS Premix (1×); and (4) CCM (control). Cells were then cultured for 21 d with media changes every 3 d. Cells were harvested for testing on days 7, 14, and 21. The harvested cells were fixed in 4% PFA in PBS, washed 2× with PBS, then stained with 1% Alcian blue solution (pH 2.5) and 0.1% Sirius red in a saturated aqueous solution of picric acid. After staining, the cells were imaged using a bright-field microscope (AmScope, Irvine, CA, USA), and the images were quantified using ImageJ (version 1.54 g).

#### 2.3.2. Induction of Chondrogenic Differentiation in GelMA-Alginate IPN Hydrogel

The cell-loaded hydrogel discs, prepared as described in Section 2.2.1, were cultured in the chondrogenic differentiation medium (CDM) containing 50 μg/mL AA2P, 40 ng/mL DXM, and 10 ng/mL TGF-β1 for 28 d with medium change every 3d. Samples were collected at the required time point for the following analysis.

#### 2.3.3. Real-Time Quantitative PCR (qRT-PCR)

Gene expression was analyzed by qRT-PCR. Total RNA was extracted from the harvested hydrogel samples using the PureLink® RNA Mini Kit (Invitroge, Thermo Fisher Scientific, Waltham, MA, USA). RNA concentration and quality were measured using a NanoDrop 2000 (Thermo Scientific, Waltham, MA, USA). Gene quantification was performed using iTaq Universal SYBR Green One-Step RT-qPCR Kit (Bio-Rad, Hercules, CA, USA) with primers for type II collagen (Col II), aggrecan (ACAN), collagen type X (Col X), SRY-box transcription factor 9 (SOX9), and glyceraldehyde 3-phophate dehydrogenase (GAPDH). The primers listed in Table 1 were synthesized by IDT (Integrated DNA Technologies) according to the sequences reported by Hamid et al., 2012 [16]. Each qRT-PCR reaction contained 5 μL iTaq Universal SYBR Green reaction mix (2×), 0.125 μL iScript reverse transcriptase, 300 nM forward primers, 300 nM reverse primers, 200 ng RNA, and nuclease-free water to a 10 μL final volume. The reaction was performed in a CFX96 instrument (Bio-Rad, Hercules, CA, USA) using the following protocol: 50 °C for 10 min (1 cycle), 95 °C for 1 min (1 cycle), followed by 40 cycles of 95 °C for 10 s each, 60 °C for 30 s (1 cycle), and completed with increments of 0.5 °C/5 s from 65 °C to 95 °C for melt curve analysis. The relative gene expression was analyzed with data normalized to GAPDH as the housekeeping gene and calculated using the 2^−ΔΔCT^ method [17].

#### 2.3.4. Immunofluorescence Staining of Type II Collagen

For immunofluorescence staining, the samples were fixed in 4% PFA for 30 min, followed by immersion in a 30% sucrose solution overnight at 4 °C. Next, the samples were embedded in Tissue-Plus™ O.C.T. Compound (FisherHealthcare, Houston, TX, USA) and sectioned at −20 °C using a cryostat (Leica). Section thickness was 20 µm. After mounting on Superfrost Plus microscope slides, the sections were permeabilized with 0.25% Triton™ X-100 for 10 min, blocked with 3% BSA at room temperature for 1 h, and labeled with Anti-Collagen II primary antibody (ab34712, Abcam, Cambridge, UK) at 1:100 overnight at 4 °C. The secondary antibody was goat anti-rabbit IgG, conjugated with Alexa Fluor^®^ 488 (Invitrogen, Thermo Fisher Scientific, Waltham, MA, USA). Secondary antibody conjugation to the primary antibody was conducted at a concentration of 4 µg/mL in PBS containing 0.2% BSA for 45 min at room temperature. Nuclei were counterstained with ProLong™ Diamond Antifade Mountant with DAPI (Invitrogen, Thermo Fisher Scientific, Waltham, MA, USA). Finally, the slides were imaged using a fluorescence microscope (Keyence).

#### 2.3.5. Alcian Blue/Nuclear Fast Red Staining of Sulphated Glycosaminoglycans (sGAGs)

The samples were fixed in 4% PFA for 30 min, dehydrated using a series of ethanol solutions ranging from 70% to 100%, cleared with xylene, embedded in Paraplast^®^ (McCormick™ Scientific, Berkeley, MI, USA), sectioned at a thickness of 7 μm using a Leica RM2245 rotary microtome, mounted on glass slides, dried at 45 °C overnight, and stored in a moisture-free environment at room temperature. Following deparaffinization in xylene and rehydration with descending 100–70% serial ethanol solutions followed by ddH_2_O, the sections were stained with 1% Alcian blue solution, pH 2.5 (Sigma-Aldrich, St. Louis, MO, USA), for 1 h then counterstained with 0.1% nuclear fast red solution (Sigma-Aldrich, St. Louis, MO, USA) for 10 min. After a final rinse in ddH_2_O, dehydration again to 100% ethanol, and clearing in xylene, the slides were mounted with Permount mounting medium (Fisher Chemical, Pittsburgh, PA, USA) and coverslipped (#1.5).

### 2.4. Evaluation of Cytokines 

#### 2.4.1. Cytotoxicity of Cytokines by MTT

To evaluate the cytotoxic effects of the cytokines (R&D Systems, Minneapolis, MN, USA) of interleukin-1 beta (IL-1β), tumor necrosis factor (TNF-α), and oncostatin M (OSM), we assessed cell viability using the MTT assay. Briefly, exponentially growing TC28a2 chondrocytes were seeded into 96-well plates (1 × 10^4^ cells/well) in CCM and allowed to grow for 24 h. The following day, the culture media for all cells was replaced with 100 μL of fresh CCM and divided in different cytokine concentration treatment groups (10, 25, 50, 100, and 200 ng/mL). After which, the cytokine-exposed treatment groups and controls were incubated for another 24 h. The wells without cytokine treatment were used as a control. At the end of the treatment, 10 μL MTT (5 mg/mL) was added to each well, then further incubated at 37 °C for 4 h. Each sample supernatant was removed by pipette and replaced with 100 μL dimethyl sulfoxide (DMSO) to dissolve the purple formazan crystals. The absorbance for each sample was then measured at 570 nm using a microplate reader (Bio Tek). The viability of cells was calculated as a percentage with the optical density (OD) value of the untreated group as the denominator. 

#### 2.4.2. Induction of MMP-13 Expression in TC28a2 Chondrocytes by Cytokines

The expression of MMP-13 in TC28a2 chondrocytes was detected using both immunofluorescences staining and the MMP-13 Human ELISA Kit (Invitrogen, Thermo Fisher Scientific, Waltham, MA, USA). Briefly, monolayer-expanded cells were treated with 25 ng/mL of IL-1β, TNF-α, and OSM, for 48 h. The supernatant from each well was collected for the ELISA, which was performed following the manufacturer’s instructions. MMP-13 concentrations were measured in duplicates and calculated from the MMP-13 standard curve generated using a 4-parameter algorithm. The monolayer of cells was fixed in 4% PFA for 30 min, washed with 2 × PBS, permeabilized with 0.25% Triton™ X-100 for 10 min, blocked with 3% BSA for 1 h, and labeled with primary antibody anti-MMP13 antibody (ab39012, Abcam, Cambridge, UK) at 1:100 overnight at 4 °C. Following this, the goat anti-rabbit IgG secondary antibody Alexa Fluor^®^ 594 (Invitrogen, Thermo Fisher Scientific, Waltham, MA, USA) was conducted at a concentration of 4 µg/mL in PBS containing 0.2% BSA for 45 min at room temperature. Nuclei were counterstained with 5 µg/mL Hoechst 33258 (Sigma-Aldrich, St. Louis, MO, USA).

### 2.5. Evaluation of MMP-13 Inhibitors

#### 2.5.1. Cytotoxicity of MMP-13 Inhibitors by MTT

To evaluate the cytotoxic effects of the MMP-13 inhibitors, the viability of TC28a2 chondrocytes was assessed using the MTT assay. Ilomastat (MedchemExpress, Monmouth Junction, NJ, USA) is a broad-spectrum matrix metalloproteinase (MMP) inhibitor. T-26c (MedchemExpress), CL-82198 (MedchemExpress), WAY-170523 (MedKoo, Durham, NC, USA), and DB04760 (MedChemExpress) are selective MMP-13 inhibitors. All inhibitors were tested at concentrations of 5, 10, 20, and 40 µM in the MTT study. TC28a2 chondrocytes were seeded into 96-well plates with 1 × 10^4^ cells per well. After overnight incubation at 37 °C, the cells were organized into individual treatment groups with each being exposed to a single MMP-13 inhibitor for 24 h. Untreated cells served as controls. Afterward, a MTT assay was performed as described in Section 2.4.1. 

#### 2.5.2. Inhibition Rate of MMP-13 Inhibitors

The inhibition rate of MMP-13 inhibitors at a concentration of 5 µM was determined by measuring comparable effects on MMP-13 activity. Briefly, Recombinant Human MMP-13 (rhMMP-13) (R&D Systems) was diluted in assay buffer (50 mM Tris, 10 mM CaCl_2_, 150 mM NaCl, 0.05% Brij-35 (*w*/*v*), and pH 7.5) to 100 µg/mL and activated by adding N-(3-Aminopropyl) methacrylamide (APMA) to achieve a final rhMMP-13 solution concentration of 1 mM. This mixture was incubated at 37 °C for 2 h. The activated rhMMP-13 was diluted to 0.2 ng/µL in assay buffer. The fluorogenic substrate for MMP, MCA-Pro-Leu-Gly-Leu-Dpa-Ala-Arg-NH_2_ (R&D Systems), was diluted to 20 µM in assay buffer. A total of 50 µL of the 0.2 ng/µL rhMMP-13 with individual MMP-13 inhibitors was added to their corresponding wells and the reaction began by adding 50 µL of the 20 µM fluorogenic substrate. The wells without MMP-13 inhibitors served as controls. After incubation of the reaction solutions at room temperature for 30 min, the fluorescence intensity (FI) was measured at excitation and emission wavelengths of 320 nm and 405 nm, respectively. The inhibition rate of the inhibitors was calculated as follows:

Inhibition rate (%) = (1 − *FI*_inhibitor_/*FI*_control_) × 100

### 2.6. Screening of MMP-13 Inhibitors Using GelMA-Alginate Cartilage Constructs with OA-like Conditions

GelMA-alginate constructs were formed as described in Section 2.2.1 and cultured in CDM for 14 d. Then, the constructs were divided into 4 groups: (1) No treatment (control); (2) 25 ng/mL IL-1β and 25 ng/mL TNF-α; (3) 25 ng/mL IL-1β, 25 ng/mL TNF-α, and 5 µM T-26c; and (4) 25 ng/mL IL-1β, 25 ng/mL TNF-α, and 5 µM Ilomastat. After treatment, these constructs were further cultured for an additional 10 d with media changes on days 3 and 7. Supernatant and constructs for all groups were harvested on days 3, 7, and 10 for subsequent analyses. Immunofluorescence staining of cryosections was performed as detailed in Section 2.3.4 and antibodies used for MMP-13 expression are described in Section 2.4.2. Type II collagen cleavage was evaluated with a C2C ELISA kit (MyBioSource, San Diego, CA, USA).

### 2.7. Histological Analysis of Human Articular Cartilage

Human cartilage specimens were sourced through the Willed Body Program at the University of North Texas (UNT) Health Science Center in Fort Worth, TX. The use of human tissue for experiments within this study was approved on 7 June 2021 by the Human Subjects Institutional Review Board (IRB) at UNT (IRB-21-337). Human articular cartilage was collected from a 60-year-old female donor with no history of osteoarthritis. The cartilage samples were harvested promptly after the donor’s passing in aseptic conditions at room temperature. The articular surface, exposed by a wide transversal arthrotomy, was macroscopically intact and smooth. The samples were fixed in 4% PFA in PBS for 24 h, then decalcified in 5% formic acid for 7 d. The samples then underwent the same processing steps as described in Section 2.3.5 before staining. H&E, Safranin O, and Alcian blue staining were then performed using standard histopathological methods commonly employed for joint tissues, as previously described [18]. For immunohistochemistry (IHC) staining of type II collagen, antigen retrieval was performed by heating the sections in sodium citrate buffer (10 mM sodium citrate, 0.05% Tween 20, and pH 6.0) at 95 °C for 30 min. Then, type II collagen was stained using a Collagen Staining Kit (Chondrex), according to the manufacturer’s instructions. Finally, the sections were developed with DAB (3,3′-Diaminobenzidine) to visualize the presence of type II collagen, resulting in a brown precipitate at the sites of antigen–antibody binding.

### 2.8. Statistical Analysis 

All experiments were conducted in triplicate, with data expressed as mean ± standard deviation (SD) using SPSS version 27.0 software (IBM, Armonk, NY, USA). Statistical comparisons were performed using one-way analysis of variance (ANOVA) with a significance level (α) set at 0.05, therefore, *p*-values of * *p* < 0.05 and ** *p* < 0.01 were defined as statistically significant. 

## 3. Results

### 3.1. Synthesis and Characterization of GelMA-Alginate IPN Hydrogel

**Figure 1:**
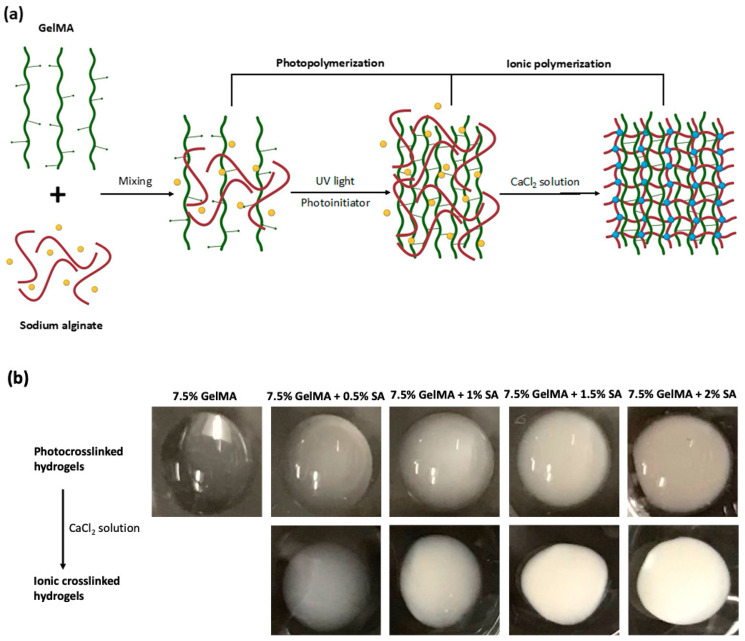
Synthesis of GelMA-alginate IPN hydrogels. (**a**) Schematic diagram of the synthesis of a GelMA-alginate IPN hydrogel. GelMA polymer chains, sodium alginate, Na^+^, and Ca^2+^ are represented by green, red, yellow, and blue colors, respectively. (**b**) Increasing opacity of GelMA-alginate IPN hydrogels corresponds to incremental increases in sodium alginate concentration.

The GelMA-alginate hydrogel was synthesized through photopolymerization and ionic polymerization of two prepolymers, GelMA and sodium alginate, as illustrated in the schematic procedure in Figure 1a. First, UV-light exposure was used to initiate the crosslinking of the GelMA component. Then, the sodium alginate component was crosslinked in the presence of calcium ions (Ca^2+^) by forming ionic bonds between the carboxylate groups and calcium ions, resulting in the crosslinking of adjacent sodium alginate chains. In this process, calcium ions effectively replace sodium ions in the alginate polymer chains, forming stable crosslinks between alginate molecules [11]. The appearance of GelMA and GelMA-alginate hydrogels differed based on their composition as shown in Figure 1b. After photocrosslinking, 7.5% GelMA had a translucent appearance. In contrast, GelMA-alginate hydrogels displayed an opaque appearance with the addition of sodium alginate, which can scatter light and reduce optical clarity. As the concentration of alginate increased in the GelMA-alginate hydrogel, the hydrogel appeared increasingly white in color. In addition, following ionic crosslinking, GelMA-alginate hydrogels exhibited a roughened or more irregular surface morphology compared to GelMA hydrogels. This roughness can be attributed to the presence of microstructures formed by sodium alginate within the hydrogel matrix.

**Figure 2:**
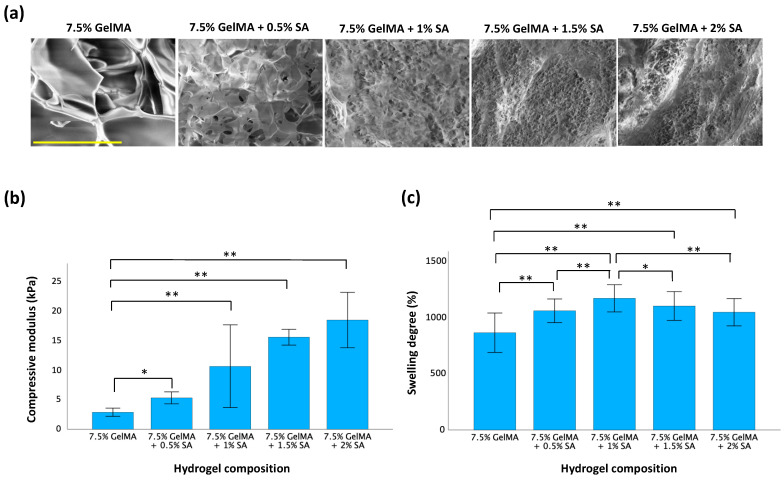
Characteristics of GelMA-alginate hydrogels with variation in sodium alginate content. (**a**) Microstructure of GelMA-alginate hydrogels. The scale bar is 100 μm. (**b**) Compressive modulus of GelMA-alginate hydrogels. (**c**) Swelling degree of GelMA-alginate hydrogels. Data are presented as mean ± SD with statistical significance indicated as * *p* < 0.05 and ** *p* < 0.01.

The specific properties of GelMA-aginate hydrogels we measured were their microstructure, compressive modulus, and swelling degree, as shown in Figure 2. The microstructure, captured by SEM, revealed that all hydrogel samples featured a porous cross-sectional morphology. Also, a highly interconnected porous structure was observed after blending with sodium alginate to form IPN hydrogels. The homogeneous distribution of the two polymer networks confirmed that the GelMA-alginate IPN hydrogel was successfully synthesized. As the concentration of sodium alginate increased, the microstructure became denser and less porous. The compressive modulus reflects the stiffness of the hydrogel. The compressive modulus of 7.5% GelMA was 2.8 kPa. However, upon incorporation of sodium alginate into GelMA, the compressive modulus increased greatly and was found to be directly proportional to the concentration of sodium alginate. Notably, the compressive modulus peaked at 18.3 kPa, a 6.5-fold increase compared to GelMA-only hydrogels, when the concentration of sodium alginate was increased to 2%. This trend demonstrated the effect sodium alginate concentration has on the stiffness of GelMA-alginate hydrogels. The swelling degree of a hydrogel reflects its ability to absorb and retain water when immersed in an aqueous solution. As the concentration of sodium alginate increased, the swelling degree of GelMA-alginate hydrogels also increased compared to GelMA-only hydrogels. However, we observed that the magnitude of increases in swelling degree gradually declined as it approached 1150% of its original weight with the 7.5% GelMA + 1% sodium alginate formulation. This suggests that, as sodium alginate concentration increases, there may be an optimal saturation point beyond which yields diminishing returns in terms of swelling capacity.

### 3.2. Cell Viability in GelMA-Alginate IPN Hydrogels

**Figure 3:**
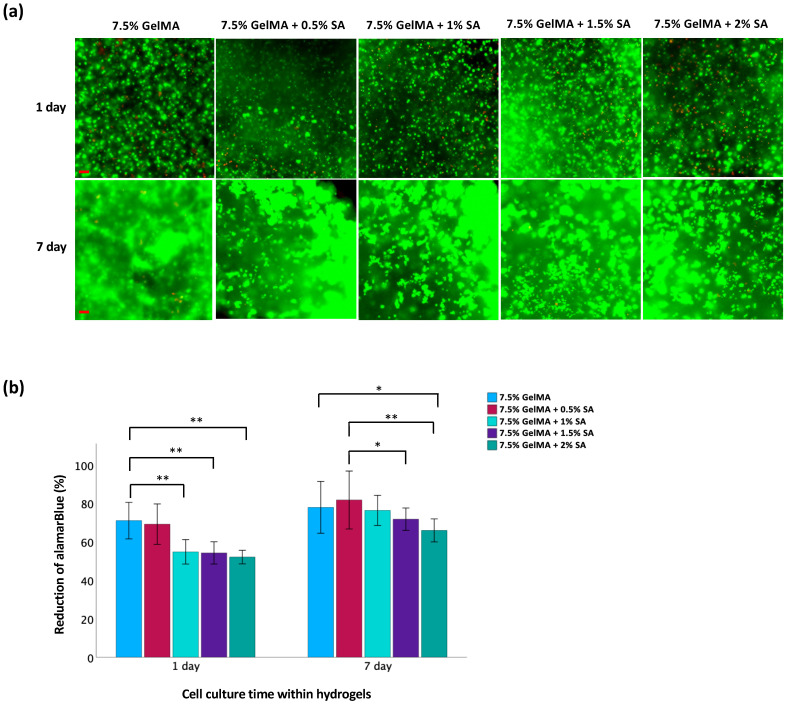
Evaluation of cell viability of TC28a2 chondrocytes in GelMA-alginate hydrogels on days 1 and 7. (**a**) Live/dead staining of chondrocytes. Green fluorescence indicates viable cells whereas red fluorescence indicates dead cells. The scale bar is 100 μm. (**b**) Metabolic activity of chondrocytes assessed by alamarBlue™ assay. Data are presented as mean ± SD, with statistical significance indicated as * *p* < 0.05 and ** *p* < 0.01.

The live/dead staining analysis revealed a high proportion of cells with green fluorescence, indicating robust viability, and minimal red fluorescence, indicative of dead cells, as illustrated in Figure 3a. Over time, a noticeable increase in live cell density was observed, accompanied by the formation of cell clusters within the hydrogel matrices. These findings suggest that the hydrogel matrices provided a conducive microenvironment for the proliferation of TC28a2 chondrocytes. However, due to differing light transmission properties between GelMA and GelMA-alginate hydrogels, fluorescence signaling alone may be unreliable when assaying the true viability of cells within these matrices. Therefore, an alamarBlue™ assay was also used to quantify cell viability (Figure 3b). This assay relies on the ability of metabolically active cells to reduce the blue-colored dye resazurin to a pink-colored compound called resorufin. The magnitude of dye reduction correlates with the metabolic activity of the cells, which is indicative of their viability [19]. The results showed no significant difference in cell viability between the 7.5% GelMA and 7.5% GelMA + 0.5% SA groups after 1 d. Also, as the concentration of SA increased in GelMA + SA groups, there was a significant decrease in cell viability compared to the 7.5% GelMA group. However, after 7 d, cell viability increased for both GelMA and GelMA + SA groups. These findings suggest that the hydrogel supports cell growth and proliferation over time. Initially, higher SA concentrations might create a more challenging environment for cells due to increased mechanical rigidity. However, as the hydrogel begins to adapt to their environment, conditions become more favorable for cell survival and proliferation. Notably, after 7 d, only the group with the highest SA concentration (2%) demonstrated a significant decrease in cell viability compared to the 7.5% GelMA group. Among the GelMA + SA groups, only the 7.5% GelMA + 0.5% SA group exhibited higher cell viability than the 7.5% GelMA + 1.5% SA and 7.5% GelMA + 2% SA groups. However, there was no significant differences observed between 7.5% GelMA + 0.5% and 7.5% GelMA + 1% groups.

### 3.3. Chondrogenic Differentiation in GelMA-Alginate IPN Hydrogels

**Figure 4:**
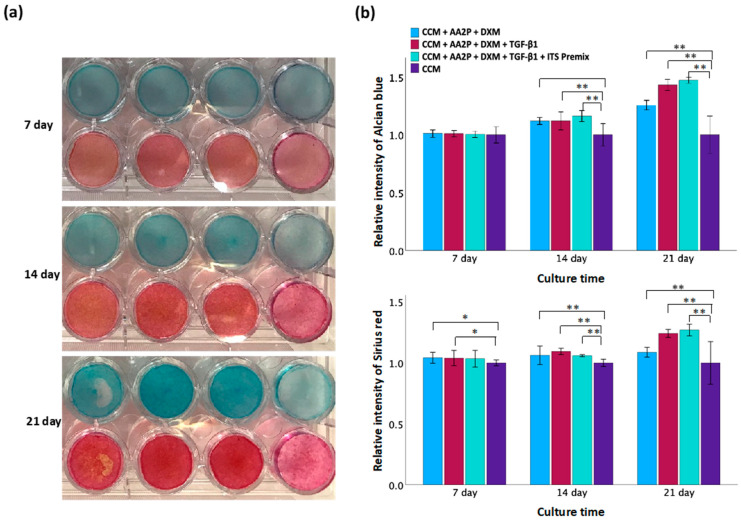
Evaluation of different chondrogenic differentiation media. (**a**) Staining of Alcian blue (upper row) and Sirius red (lower row) on days 7, 14, and 21. The culture medium in each group from left to right is CCM + AA2P + DXM, CCM + AA2P + DXM + TGF-β1, CCM + AA2P + DXM + TGF-β1 + ITS Premix, and CCM only (control). (**b**) Quantification of Alcian blue staining and Sirius red staining. Data are presented as mean ± SD with statistical significance indicated as * *p* < 0.05 and ** *p* < 0.01.

To identify the optimal differentiation medium for TC28a2 chondrocytes, various combinations of supplements were evaluated (Figure 4). Chondrogenic differentiation was confirmed by Alcian blue staining for GAG and Sirius red staining for collagen. Following a 14 d differentiation period, a greater staining intensity was observed within the treated groups compared to controls. At the end of the 21 d differentiation, period staining became more pronounced, particularly in groups treated with CCM + AA2P + DXM + TGF-β1 and CCM + AA2P + DXM + TGF-β1 + ITS Premix. However, no significant difference in staining intensity was observed between these two groups when compared directly. These results suggest that the medium supplemented with AA2P, DXM, and TGF-β1 was effective in promoting chondrogenic differentiation.

**Figure 5:**
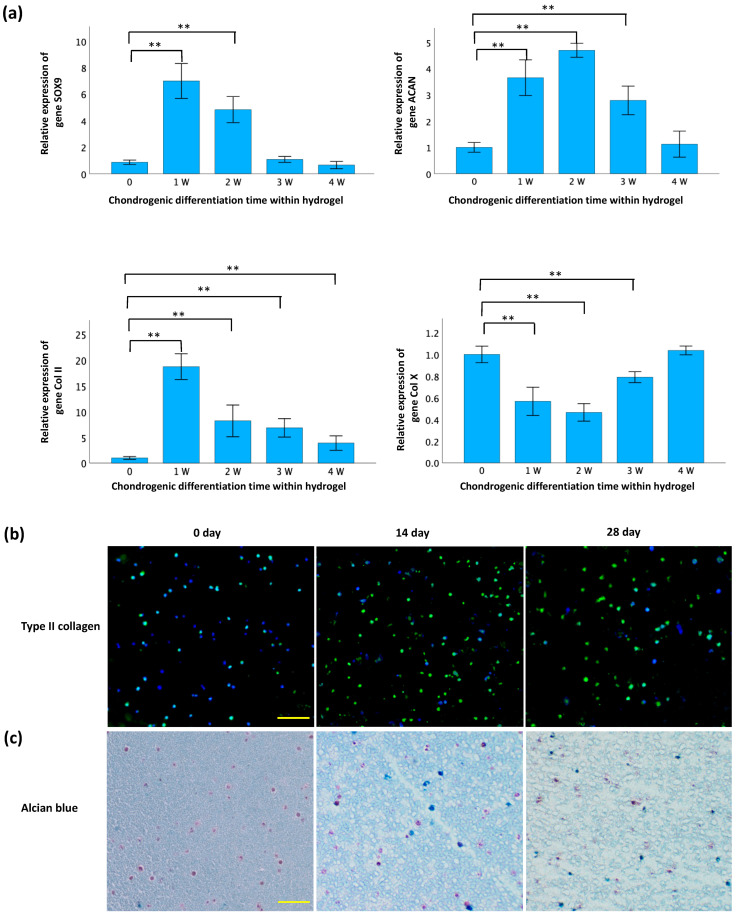
Confirmation of chondrogenesis in GelMA-alginate hydrogel. (**a**) qRT-PCR analysis of gene expression of chondrogenic markers. Gene expression is normalized to GAPDH and expressed relative to the control group (Day 0). Data are presented as mean ± SD with statistical significance indicated as * *p* < 0.05 and ** *p* < 0.01. (**b**) Immunofluorescence staining of type II collagen (green) in cryosections. The scale bar is 100 μm. (**c**) Alcian blue/nuclear fast red staining of sulfated glycosaminoglycans (sGAGs) in paraffin sections. Nuclei, cytoplasm, and sGAGs are stained dark pink to red, pale pink, and blue, respectively. The scale bar is 100 μm.

The validation of differentiation of TC28a2 chondrocytes within GelMA-alginate hydrogels is presented in Figure 5. Following the initiation of differentiation, a notable increase in the expressions of SOX9, ACAN, and Col II genes was observed (Figure 5a). However, the increase in SOX9 expression was not statistically significant after 14 d and the increase in ACAN expression was not significant after 21 d. In contrast, a significant upregulation in Col II expression persisted for 28 d. Furthermore, the expression of catabolic gene Col X significantly decreased for 14 d, then gradually increased to levels prior to differentiation. Figure 5b shows an increase in immunofluorescence intensity of type II collagen staining after differentiation, indicating higher levels of this protein within the chondrocytes. In the Alcian blue staining (Figure 5c), the nuclei were stained dark pink to red, cytoplasm was stained pale pink, and glycosaminoglycans (GAGs) were stained blue. The results show a significant abundance of GAGs deposited around cells after differentiation. These results suggest that the hydrogel environment is conducive to promoting chondrogenesis in these cells using the chondrogenic differentiation medium.

### 3.4. Evaluation of Cytokines for MMP-13 Induction 

**Figure 6:**
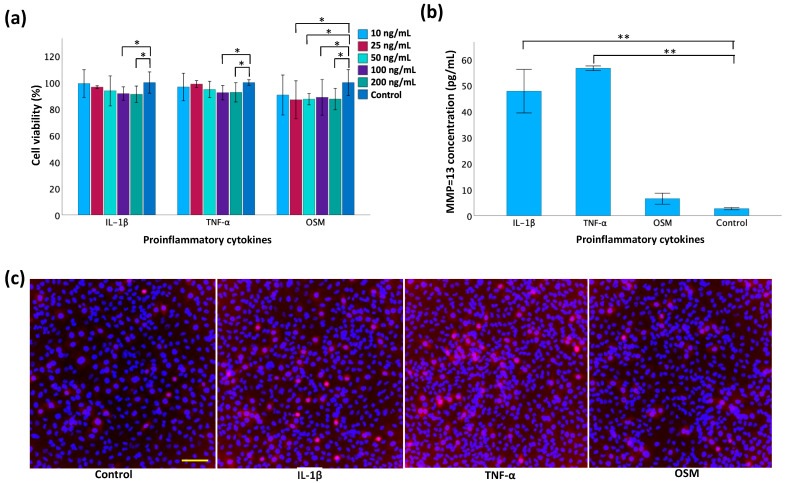
Evaluation of cytokines for MMP-13 induction in monolayer TC28a2 chondrocytes. (**a**) Cell viability using MTT assay 24 h post-treatment with inflammatory cytokines. (**b**) Measurement of MMP-13 concentration by ELISA in cell supernatant 2 d post-cytokine induction. (**c**) Immunofluorescence staining of MMP-13 2 d post-cytokine induction. The scale bar is 100 μm. Data are presented as mean ± SD with statistical significance indicated as * *p* < 0.05 and ** *p* < 0.01.

In the MTT assay (Figure 6a), a series of concentrations for each cytokine—TNF-α, IL-1β, and OSM—were tested on the TC28a2 chondrocytes to evaluate their individual effects on cell viability. A significant increase in cell death became apparent when the concentration reached 100 ng/mL for IL-1β and TNF-α, and 25 ng/mL for OSM. This indicated the concentrations at which these cytokines are toxic to TC28a2 chondrocytes. For the expression of MMP-13, ELISA analysis (Figure 6b) demonstrated a significant increase in MMP-13 concentration in the cell culture supernatant following treatment for both IL-1β and TNF-α compared to the non-treated controls. Immunofluorescence staining (Figure 6c) revealed a distinct increase in red fluorescence signal intensity, indicating MMP-13 expression in the cytokine-treated groups compared to the controls. Additionally, the fluorescent intensity was greater in the TNF-α and IL-1β groups than in the OSM-treated group. 

### 3.5. Evaluation of MMP-13 Inhibitors 

**Figure 7:**
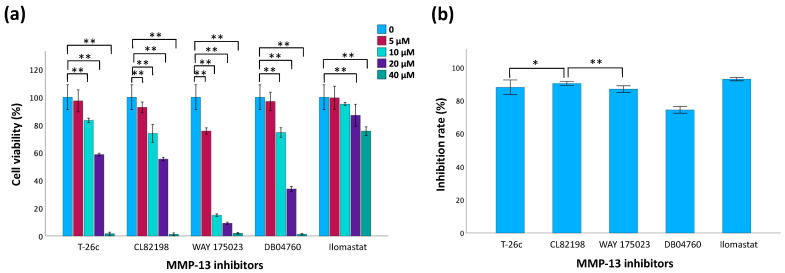
Evaluation of MMP-13 inhibitors. (**a**) Cell viability of TC28a2 chondrocytes using MTT assay 24 h post-treatment with MMP-13 inhibitors. (**b**) Comparison of inhibitory effects of inhibitors using a fluorogenic substrate assay. Data are presented as mean ± SD with statistical significance indicated as * *p* < 0.05 and ** *p* < 0.01.

The MTT assay results (Figure 7a) suggest that cell viability decreases as the concentration of inhibitors increases, indicating that inhibitor toxicity increases with concentration. Compared to the controls, no significant difference in cell viability was observed for the 5 µM T-26c and 10 µM Ilomastat treatment groups. The inhibition rate, represented as the percentage of MMP-13 activity inhibited by the inhibitors, was also measured (Figure 7b). The broad-spectrum inhibitor Ilomastat demonstrated the greatest inhibitory effects on MMP-13 activity. Also, among the selective MMP-13 inhibitors, CL82198 exhibited a higher inhibition rate compared to others. Considering the results of both cell viability and inhibition rate assays, it is important to select inhibitors with low cellular toxcicity and exhibit significant inhibition of MMP-13 activity. Consequently, 5 µM T-26c and 5 µM Ilomastat were the only inhibitors chosen for subsequent experiments.

### 3.6. Screening of MMP-13 Inhibitors Using GelMA-Alginate Cartilage Constructs with OA-like Conditions

**Figure 8:**
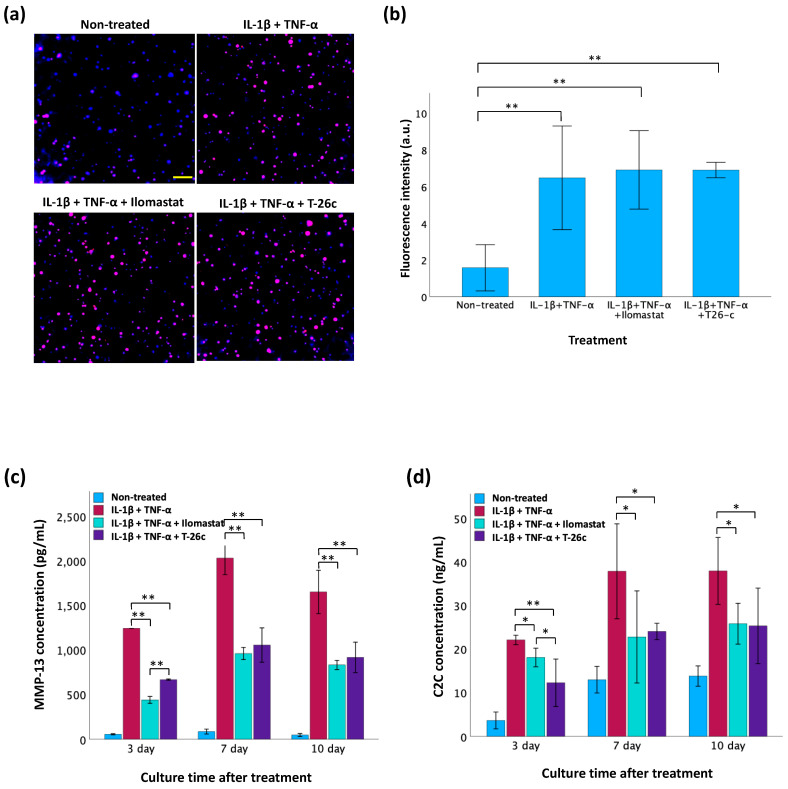
Screening of MMP-13 inhibitors using GelMA-alginate cartilage constructs with OA-like conditions. (**a**) Evaluation of MMP-13 expression in TC28a2 chondrocytes using immunofluorescence staining 3 d post-treatment with IL-1β and TNF-α. The scale bar is 100 μm. (**b**) Fluorescence intensity of MMP-13 expression in TC28a2 chondrocytes analyzed by ImageJ (version 1.54 g). (**c**) Measurement of active MMP-13 concentration by ELISA in cell supernatant. (**d**) Measurement of C2C concentration for type II collagen cleavage by ELISA in cell supernatant. Data are presented as mean ± SD with statistical significance indicated as * *p* < 0.05 and ** *p* < 0.01.

In our modeling of OA conditions with GelMA-alginate cartilage constructs (Figure 8a,b), the fluorescence intensity of MMP-13 was significantly elevated in the IL-1β- and TNF-α-treated groups. However, there was no significant difference in the expression of MMP-13 among the inhibitor-treated groups and the cytokine-only group. These results indicate that IL-1β and TNF-α biochemically induces the inflammatory OA model in vitro. The ELISA results (Figure 8b) reveal the MMP-13 concentration decreases in the inhibitor-treated groups, suggesting that the activity of MMP-13 in the supernatant is inhibited. In addition, the Ilomastat displayed greater inhibition compared to T-26c. The C2C fragment, generated through the degradation of type II collagen by enzymes, such as MMP-13 [20], exhibited elevated levels in correlation with MMP-13 activity as confirmed by the ELISA results in Figure 8c. Increased MMP-13 concentration post-incubation with cytokines corresponded to higher C2C concentrations, indicating greater type II collagen cleavage. Conversely, inhibitors in the culture medium led to a reduced MMP-13 concentration, resulting in diminished type II collagen cleavage. Notably, no significant difference was observed between the two inhibitors throughout the duration of the experiment.

### 3.7. Histological Analysis of Human Articular Cartilage

**Figure 9:**
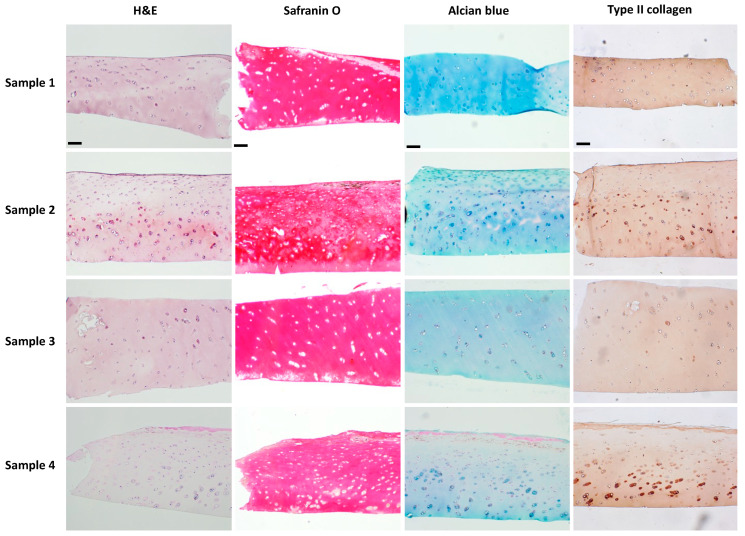
Histological analysis of human articular cartilage by staining of H&E, Safranin O, and Alcian blue, and IHC staining of type II collagen. The scale bar is 100 μm.

The results of human articular cartilage staining are shown in Figure 9. In H&E staining, chondrocytes are typically round or ovoid in shape with a nucleus surrounded by a small amount of cytoplasm. They are isolated within lacunae, which are small cavities or spaces within the cartilage matrix. Chondrocytes in the outermost layer were flattened and aligned parallel to the surface of the cartilage as observed in samples 2 and 4. However, this alignment was absent in samples 1 and 3, suggesting the superficial layer’s absence. Typically, chondrocyte nuclei appear blue-purple, while cytoplasm appears pink or faintly stained based on eosin intensity. Notably, in sample 2, some chondrocytes displayed a purple-stained cytoplasm, indicating cellular hypertrophy, a feature often associated with pathological conditions like osteoarthritis. Safranin O and Alcian blue staining are commonly used to visualize cartilage matrix components. Safranin O selectively binds to acidic proteoglycans, staining them red, while Alcian blue selectively binds to sulfated glycosaminoglycans (sGAGs), staining in blue. In healthy cartilage, both stains yield intense and uniform staining of proteoglycans and sGAGs, respectively, throughout the ECM. However, our staining patterns appeared patchy with a locally reduced intensity, indicating a loss or depletion of proteoglycans and sGAGs associated with cartilage degeneration or pathology. The results of the IHC staining of type II collagen appear as brown. Uniform and continuous staining throughout the tissue may indicate healthy and intact cartilage. However, this staining also appeared patchy and discontinuous, also suggesting areas of cartilage degradation or disease.

## 4. Discussion

The synthesis of GelMA-alginate IPN hydrogels typically involves the simultaneous polymerization of GelMA via photo-initiated crosslinking and the gelation of sodium alginate through ionic interactions. The formation of interpenetrating polymer networks combines the desirable properties of both GelMA and sodium alginate, resulting in hydrogels with enhanced mechanical properties, which are more biologically accommodating. GelMA is widely utilized for its incorporation of the RGD (arginine–glycine–aspartate) peptide motif. This tripeptide sequence serves as a cell adhesion ligand by mimicking natural cell-binding domains found in extracellular matrix proteins, like fibronectin and collagen [21]. This is crucial for maintaining cell viability and functionality in 3D culture systems. However, GelMA hydrogels typically have lower mechanical strength compared to synthetic hydrogels, which may limit their utility in load-bearing applications or tissue engineering constructs requiring high mechanical integrity [22]. Incorporating sodium alginate into the GelMA formulation can help overcome this limitation. Sodium alginate molecules act as reinforcements within the GelMA matrix, providing additional structural integrity and resistance to deformation, consequently leading to a stiffer hydrogel. By blending GelMA with sodium alginate, we can create composite hydrogels that combine the bioactivity of GelMA with the mechanical robustness of sodium alginate.

Sodium alginate is a hydrophilic polymer with a high affinity for water due to its hydrophilic hydroxyl and carboxyl groups [13]. When sodium alginate is incorporated into GelMA, it increases the hydrophilicity of the hydrogel network, promoting water absorption and swelling. Usually, higher concentrations of sodium alginate provide more sites for water absorption and can lead to increased swelling degree. However, the ability of water molecules to penetrate the hydrogel network is also influenced by the crosslinking density, which affects the mesh size and porosity [23]. Lower crosslinking density allows for greater swelling as there are fewer crosslinks restricting the movement of polymer chains and water molecules. Conversely, higher crosslinking density reduces the swelling capacity due to the increased number of crosslinks. This phenomenon results in a more compact and less porous network structure making it challenging for water molecules to penetrate and effectively swell the hydrogel. This may explain why the swelling degree decreased as the concentration of sodium alginate increased from 1% to 1.5% to 2% (Figure 2c). Therefore, it is necessary to characterize the specific swelling profile of each hydrogel formulation under the desired conditions. 

The water absorption capacity and mechanical strength of a hydrogel can influence cell viability within the hydrogel matrix. A hydrogel with a high-water-absorption capacity can retain a larger volume of culture medium, more readily facilitating the diffusion of nutrients and oxygen to encapsulated cells [24]. Adequate nutrient and oxygen supply is crucial for cell metabolism and viability. Additionally, hydrogels with higher compressive strength provide better mechanical support to encapsulated cells. This mechanical support can prevent cell deformation or damage under physiological loading conditions, further promoting cell viability. However, hydrogels with very high compressive strength and denser structure may deform cells and impede the diffusion of nutrients, reducing cell viability. This is evidenced by the results shown in Figure 2 and Figure 3. As the concentration of sodium alginate increased, the compressive stress increased, but the cell viability significantly decreased at sodium alginate concentrations of 1.5% and 2% compared to the GelMA-only group. Therefore, an optimal balance of swelling degree and mechanical strength in hydrogels is essential for fostering favorable conditions that support cell viability. In this study, the IPN GelMA-alginate hydrogel, synthesized from 7.5% GelMA and 1% sodium alginate, demonstrated comparable cell viability to the 7.5% GelMA hydrogel but with a superior mechanical performance. Consequently, the hydrogel comprising 7.5% GelMA and 1% sodium alginate formulation was chosen for subsequent experiments.

TC28a2 chondrocytes, a commonly used cell line derived from human articular cartilage and established by Mary B. Goldring [25], are terminally differentiated cells with limited potential to exhibit chondrogenic characteristics. This includes the expression of cartilage-specific markers, such as SOX9, type II collagen, and aggrecan [26]. This specialized state enables them to transiently upregulate marker genes under chondrogenic differentiation conditions. Despite this short-term differentiation, TC28a2 chondrocytes remain a suitable choice for inducing an in vitro OA model due to their acute sensitivity to inflammatory stimuli. This allows for the timely investigation of key events in OA pathogenesis, such as the upregulation of catabolic factors like MMP-13 and the degradation of cartilage matrix components.

The in vitro OA model based on GelMA-alginate cartilage constructs can be used to test the effectiveness of MMP-13 inhibitors. After stimulation with IL-1β and TNF-α, MMP-13 fluorescence intensity was significantly enhanced, as shown in Figure 8, indicating that the cytokines effectively simulate the inflammatory conditions characteristic of OA. However, in all IL-1β- and TNF-α-treated groups both with and without inhibitors, there was no significant difference in MMP-13 fluorescence intensity. This can be explained by the fact that MMP-13 is synthesized in an inactive form (proMMP-13) in the chondrocytes, then secreted into the ECM, where it is activated by other proteases [7]. Consequently, MMP-13 inhibitors are designed to target the active site of the enzyme that is not available for binding until after MMP-13’s post-translational modification, which occurs outside of the cell. The C2C fragment, a marker of type II collagen degradation, was elevated in correlation with MMP-13 activity. Higher MMP-13 concentrations due to cytokine treatment led to increased C2C levels, reflecting greater collagen breakdown. The presence of inhibitors resulted in reduced MMP-13 concentration and subsequently lower C2C levels, demonstrating their efficacy in diminishing type II collagen cleavage. Despite the overall inhibition of MMP-13 activity by both pan-inhibitor Ilomastat and selective inhibitor T-26c, no significant difference was observed between them. Ilomastat can inhibit all the collagenases (MMP-1, MMP-8, and MMP-13) involved in the degradation of type II collagen, while T-26c only inhibits MMP-13. This indicates that MMP-13 is the main enzyme induced by cytokines. In summary, this 3D in vitro model provides a rapid, reproducible, and reliable model system to study the mechanisms of cartilage degradation and the potential therapeutic effects of its inhibitors.

Cartilage explants retain the native tissue architecture and cellular composition found in vivo, allowing researchers to study OA-related processes in a more physiologically representative context. However, maintaining sample consistency is challenging. For instance, Figure 9 illustrates how, even when sourced from the same individual, cartilage tissue degradation can vary considerably across different regions **of** the joint. This inherent variability complicates efforts to ensure uniformity in experimental samples. Furthermore, the phenotypic heterogeneity expressed among individuals introduces an additional layer of complexity when comparing samples. In contrast, hydrogel-based cartilage constructs serve as promising alternatives to cartilage explants for their consistent composition. These constructs offer a more standardized model compared to the innate variability of cartilage explants. Moreover, hydrogel-based platforms can be adapted for high-throughput screening of potential drug candidates, therapeutics, or biomaterials, providing a more efficient and scalable approach compared to traditional cartilage explants.

## 5. Conclusions

In conclusion, the synthesis of GelMA-alginate IPN hydrogels offers a flexible platform for cartilage tissue engineering, given how readily its mechanical and biological properties may be modified. By combining GelMA with sodium alginate, we can create composite hydrogels that feature both the bioactivity of GelMA and enhanced mechanical stiffness. Furthermore, sodium alginate’s hydrophilic nature increases water absorption and swelling within the hydrogel network, a function crucial for nutrient and oxygen diffusion to support cell viability. However, the swelling behavior of hydrogels is influenced by factors such as crosslinking density and polymer concentration, necessitating mindful consideration in hydrogel design.

The GelMA-alginate IPN hydrogel synthesized from 7.5% GelMA and 1% sodium alginate provided a suitable environment for TC28a2 chondrocytes to develop cartilage constructs with the chondrogenic differentiation medium composed of CCM + 50 μg/mL AA2P + 40 ng/mL DXM + 10 ng/mL TGF-β1. This 3D structure could replicate the complex architecture and microenvironment of tissues compared to traditional 2D cultures. In addition, the 3D OA model allowed for consistent and replicable drug screenings. In contrast, cartilage explants offer a more physiologically relevant model, but pose challenges in maintaining sample consistency and homogeneity across individuals.

Overall, hydrogel-based cartilage constructs represent a promising alternative to cartilage explants, offering consistent composition, standardization of models, and scalability for high-throughput screening of potential therapeutics. These advantages in hydrogel-based tissue engineering hold significant promise for advancing our understanding and treatment of osteoarthritis and other cartilage-related disorders.

## Figures and Tables

**Table 1:** Primers used for qRT-PCR analysis.

Target Gene	Accession Number	Forward Primer (5′–3′)	Reverse Primer (3′–5′)
Col II	NM_001844 (https://www.ncbi.nlm.nih.gov/nuccore/NM_001844)	CTATCTGGACGAAGCAGCTGGCA	ATGGGTGCAATGTCAATGATGG
Col X	NM_000493 (https://www.ncbi.nlm.nih.gov/nuccore/NM_000493)	GCTAAGGGTGAAAGGGGTTC	CTCCAGGATCACCTTTTGGA
ACAN	NM_001135 (https://www.ncbi.nlm.nih.gov/nuccore/NM_001135)	CACTGTTACCGCCACTTCCC	ACCAGCGGAAGTCCCCTTCG
SOX9	NM_000346 (https://www.ncbi.nlm.nih.gov/nuccore/NM_000346)	GCGGAGGAAGTCGGTGAAGA	CCCTCTCGCTTCAGGTCAGC
GAPDH	NM_002046 (https://www.ncbi.nlm.nih.gov/nuccore/NM_002046)	TCCCTGAGCTGAACGGGAAG	GGAGGAGTGGGTGTCGCTGT

## Data Availability

The data presented in this study are available on request from the corresponding author. The data are not publicly available due to ongoing analysis, the sensitive nature of some samples, and the data size.
